# Supplementary material for: Poor WASH (Water, Sanitation, and Hygiene) Conditions Are Associated with Leprosy in North Gondar, Ethiopia
Source: Int J Environ Res Public Health. 2020 Aug 20;17(17):6061. doi: 10.3390/ijerph17176061 (PMC7504265; doi:10.3390/ijerph17176061)
Supplement: Supplementary file 1 [file ijerph-17-06061-s001.pdf]

Supplementary Material. Study Questionnaire

Hi my name is __________. Thank you for participating in this survey. We are part of a team from Emory University and University of Gondar and are interested in reducing the number of infections in your home and community. Your answers will provide valuable knowledge that will help protect your home and community. You have signed a consent form and everything you say will be kept private and anonymous. Your participation is completely voluntary, and you are allowed to change your mind at any point in time. However, we appreciate your contribution. This survey should take approximately 20 minutes. Do you still agree to participate?

Yes No

Part. A. Demographic and Household Information

Question:	Response:
A1. How old were you, in years, on your last birthday?	_______<br><br>Refused………..98<br>Didn't Answer……..99
A2. What is your gender?	Male………1<br>Female…….2<br><br>Refused…………98<br>Didn't Answer……….99
A3. How many people live in your home?	[___ ___]<br><br>Refused…………..…..98<br>Don't know………….99
A4. Does any member in this household own[1]<br>A6a. A watch?<br>A6b. A bicycle?<br>A6c. A motorcycle or motor scooter?<br><br>A6d. An animal-drawn cart?	Yes No<br>a. Watch……..……....1 2<br>b. Bicycle………...….1 2<br>c. Motorcycle /<br>scooter…………….….1 2<br>d. Animal-drawn<br>cart ………………....1 2

A6e. A car or truck?<br>A6f. A boat with motor?<br>A6g. A mobile phone?<br>A6h. A radio?	e. Car or truck……...1 2<br>f. Boat with motor..1 2<br>g. A mobile phone ..1 2<br>h. A radio……………..1 2
A5. Does any member of this household own agricultural land?[1]	a. Yes………………………………….………1<br>b. No…………………………………….2<br>c. Refused……………………………98<br>d. Don't know………………………99
A6. Does any member of this household own any livestock, herds, other farm animals, or poultry?[1]	a. Yes…………………………….……….1<br>b. No…………………………………...2<br>c. Refused…………………………98<br>d. Don't know……………………..99
A7. Does any member of this household have a bank account?[1]	a. Yes…………………………….……….1<br>b. No………………………………..…..2<br>c. Refused………………………… 98<br>d. Don't know……………………..99
A8. What grade level did you complete?	a. [____ ____]<br>b. None………………………………..0<br>c. Refused…………….………….98<br>d. Don't know…………………..…99

Part B. Household Water and Hygiene

Question:	Response:
B1. What is the MAIN source of drinking water for members of your household?[2]<br>*Please only state one.*	a. Piped water to household……11<br>b. Piped water to yard…………….12<br>c. Public tap / standpipe………...13<br>d. Borehole…………………………...14<br>e. Protected dug well……………..15<br>f. Unprotected dug well………..21<br>g. Protected spring………………...16<br>h. Unprotected spring……………22<br>i. Rainwater collection…………..17<br>j. Bottled water……………………..18<br>k. Cart with small tank / drum..23<br>l. Tanker-truck……………………...24<br>m. Surface water……………………25<br>n. Other…………………………………88

	Specify____________________<br>o. Refused……………………………..98<br>p. Don't know………………………..99
B2. What is the MAIN source of water used for cooking?[2]<br>*Please only state one.*	a. Piped water to household……11<br>b. Piped water to yard…………….12<br>c. Public tap / standpipe………...13<br>d. Borehole…………………………….14<br>e. Protected dug well……………...15<br>f. Unprotected dug well………...21<br>g. Protected spring………………...16<br>h. Unprotected spring……………22<br>i. Rainwater collection…………..17<br>j. Bottled water………………….….18<br>k. Cart with small tank / drum..23<br>l. Tanker-truck……………………...24<br>m. Surface water……………….….25<br>n. Other………………………………….88<br>Specify____________________<br>o. Refused…………………………..…..98<br>p. Don't know…………………….…..99
B3. What is the MAIN source of water used for bathing and hand washing?[2]<br>*Please only state one.*	a. Piped water to household……11<br>b. Piped water to yard…………….12<br>c. Public tap / standpipe………...13<br>d. Borehole…………………………….14<br>e. Protected dug well……………..15<br>f. Unprotected dug well…………21<br>g. Protected spring………………...16<br>h. Unprotected spring……………22<br>i. Rainwater collection…………..17<br>j. Bottled water……………………..18<br>k. Cart with small tank / drum..23<br>l. Tanker-truck………………………24<br>m. Surface water…………………….25<br>n. Other…………………………………88<br>Specify____________________<br>o. Refused……………………………..98<br>p. Don't know………………………...99

B4. How long does it take (in minutes) to go to the water source, to get drinking water, and come back?[2]	a. Number of minutes___ ___ ___<br>b. Refused……………………………….98<br>c. Don't know……………………..……99
B5. Who collects the water the most frequently?	a. The interviewee…………….…….1<br>b. Child – age ___ ___ ………………..2<br>c. Spouse………………………………..3<br>d. Other adult in the household..4<br>e. Other…………………………………88<br>f. Refused……………………………..98<br>g. Don't know………………………..99
B6. Do you treat your water in any way to make it safer to drink?[2]	a. Yes…………………………......…1 → B7<br>b. No……………………………...…2 → B8<br>c. Refused……………..……..…98 → B8<br>d. Don't know…………..…..….99 → B8
B7. What do you usually do to the water to make it safer to drink?[2]<br>*Please state only one.*	a. Boil……………………………….11<br>b. Add bleach / chlorine………12<br>c. Strain it through a cloth…...21<br>d. Use a water filter……………..13<br>e. Solar disinfection…………….14<br>f. Let it stand and settle……….22<br>g. Other……………………………….88<br>Specify____________________<br>h. Refused……………………………..98<br>i. Don't know………………………..99
B8. What type of toilet facility do the members of your household most often use?[2]<br>*Please state only one.*	a. Flush toilet………………………11<br>b. Ventilated improved pit latrine…………………………....12<br>c. Pit latrine with slab………………………………..13<br>d. Pit latrine without slab / open pit……………………………….………21<br>e. Composting toil………………..22<br>f. Bucket…………………………….23<br>g. Hanging toilet…………….….24<br>h. No facilities or fi ………………29<br>i. Other ……………………………...88<br>Specify_________________________<br>j. Refused…………………………...98<br>k. Don't know………………………99
B9. Do you share this facility with other households?[2]	a. Yes ………………………………..1 → B10<br>b. No ………………………………..2 → B11<br>c. Refused……………………..…98 → B11<br>d. Don't know………..……………99 →B11
B10. How many households use this facility?[2]	a. [___ ___ ___]<br>b. Refused……………………………98<br>c. Don't know….………………..…….99

B11. Do you have any children who are not toilet-trained?	a. Yes…………………………...………1 → B12<br>b. No……………………………......2 → C1<br>c. Refused…………………………98 → C1<br>d. Don't know…………………...99 → C1
B12. The last time your youngest child had a bowel movement, what was done to dispose of the stools?[2]	a. Child used latrine / toilet……1<br>b. Rinsed / placed into toilet / latrine………………………………..2<br>c. Rinsed / placed into drain or ditch………………………………….3<br>d. Thrown into garbage…………..4<br>e. Buried………………………………..5<br>f. Left in the open…………………..6<br>g. Other……………..........................88<br>Specify________________________<br>h. Refused……………………………98<br>i. Don't know..……………………..99

Part C. Knowledge of Infection Transmission

Question:	Response:
C1. How does one get infected with intestinal worms?	a. Contaminated food…………………...1<br>b. Contaminated water…………………2<br>c. Mosquitos……………………………...3<br>d. Swimming……………………………..4<br>e. Animals……………………………….….5<br>f. Personal contact with others…,…6<br>g. Walking barefoot……………………..7<br>h. Other (specify)________________.....88<br>i. Refused……………………………….…98<br>j. Don't know………………………….…99
C2. What can be done to prevent infection with intestinal worms?	a. Treating drinking water…….……1<br>b. Washing hands…………….……….…2<br>c. Toweling off……………………………3<br>d. Wearing shoes……………………..….4<br>e. Defecating in a latrine……………..…5<br>f. Avoid swimming……………………….6<br>g. Cook food thoroughly……………...7<br>h. Take pills………………………………..8<br>i. Other ………………………………….…..88<br>Specify____________________________

	j. Refused………………………………....98<br>k. Don't know…………………………...99
C3. What causes Bilharzia (schistosomiasis)?	a. Contaminated food…………….….1<br>b. Contaminated water………………..2<br>c. Mosquitos…………………………….3<br>d. Swimming in fresh water…………4<br>e. Animals………………………………..5<br>f. Personal contact with other..…...6<br>g. Walking barefoot…………………....7<br>h. Snails……………………………..….8<br>i. Other ………………………………….88<br>Specify ______________________________<br>j. Refused………………………………....9<br>k. Don't know…………………………...99
C4. What can be done to prevent infection with schistosomiasis?	a. Treating drinking water……..……1<br>b. Washing hands………….……….....2<br>c. Toweling off…………………...…..3<br>d. Wearing shoes……………….….….4<br>e. Defecating in a latrine……….….….5<br>f. Avoid swimming……………………..6<br>g. Avoid urinating in fresh water…7<br>h. Other …………………………………88<br>Specify______________________________<br>i. Refused………………………………….98<br>j. Don't know……………………………99
C5. How do you catch leprosy/Hansen's Disease?	a. Drinking "bad" water……...………1<br>b. Not washing your hands……..…..2<br>c. Eating "bad "food………...…..…….3<br>d. From the air/inhaling…………....4<br>e. From animals………………….……..5<br>f. Touching someone with leprosy………………………..……...6
C6. How do you protect yourself from leprosy/Hansen's Disease?	a. Washing your hands……………..…..1<br>b. Good personal hygiene…………...2<br>c. Using soap when bathing……....3<br>d. Washing your clothes …………....4<br>e. Washing your face……………….....5

Part E. Handwashing

Questions:	Responses:
E1. Where do you wash your hands at home?	a. No handwashing……………………………1<br>b. In kitchen…….……………………………………2<br>c. By latrine/toilet…………………………...…3

	d. Other (please specify)______________________<br>e. Refused to answer…………………………4<br>f. Don't know……………………………………..5
E2. Do you have soap available today to wash hands at home?	a. Yes……………………………………1<br>b. No………………………..………..2<br>c. Refused…………………….………98<br>d. Don't know……………...…..…99

Thank you for participating in this survey Your time and contribution will increase knowledge to fight infection in your community.

End time __ __: __ __
